# Outbreak of Dengue and Chikungunya Fevers, Toamasina, Madagascar, 2006

**DOI:** 10.3201/eid1407.071521

**Published:** 2008-07

**Authors:** Mahery Ratsitorahina, Julie Harisoa, Jocelyn Ratovonjato, Sophie Biacabe, Jean-Marc Reynes, Hervé Zeller, Yolande Raoelina, Antoine Talarmin, Vincent Richard, Jean Louis Soares

**Affiliations:** * Institut Pasteur de Madagascar, Antananarivo, Madagascar; † Direction Provinciale de la Santé, Toamasina, Madagascar; ‡ Institut Pasteur, Lyon, France; § Direction des Urgences et de la Lutte contre les Maladies Transmissibles, Antananarivo

**Keywords:** Madagascar, dengue virus, chikungunya virus, disease outbreaks, Aedes albopictus, dispatch

## Abstract

An outbreak of dengue-like syndrome occurred in Toamasina from January through March 2006. Dengue type l or chikungunya viruses were detected in 38 of 55 patients sampled. *Aedes albopictus* was the only potential vector collected. Of 4,242 randomly selected representative residents interviewed retrospectively, 67.5% reported a dengue-like syndrome during this period.

Arbovirus infections, arthropod-transmitted viral diseases, are common health risks in tropical and subtropical areas. Since early 2006, chikungunya fever, a crippling mosquito-borne disease, has emerged in the southwestern nations of the Indian Ocean. An epidemic started in Kenya in 2004 and in Moroni (Comoros Island) early in 2005 (*1*). Increasing incidence of the disease was first reported in April 2005 in the French island of Réunion. Approximately 5,000 cases were reported up to December, when a massive epidemic began (*2*). By the first week of March 2006, the virus had spread to the islands of Seychelles, Mauritius, and Mayotte (*1*).

During that same period, in January 2006, an outbreak of denguelike syndrome (DLS) was reported in Toamasina, on Madagascar’s east coast. The results of the virologic and entomologic investigations are reported below. A retrospective cross-sectional study was also conducted to assess the extent of the outbreak.

## The Study

Laboratory investigation began when the alert was given in January 2006. Serum samples were obtained from patients who had fever lasting <5 days and at least 3 of the following symptoms: headache, myalgia, arthralgia, retroorbital pain, or rash. Serum samples were sent from the health centers in Toamasina to the Institut Pasteur de Madagascar in Antananarivo in dry nitrogen containers and then stored at –80°C. Altogether, 55 patients were sampled; second samples were obtained from 14 patients. RNA extraction and chikungunya virus (CHIKV) detection were performed as previously described (*1*). Dengue virus (DENV) detection was performed according to the technique of Lanciotti et al. (*3*). Immunoglobulin (Ig) M and IgG against CHIKV and DENV were detected by ELISA as previously described (*4*).

Entomologic investigation was conducted February 14–17, 2006, to determine the abundance of DENV and CHIKV in urban mosquito vectors. Five neighborhoods in which human cases of DLS had been reported were explored. Mosquito larvae collected by manual method at all potential larvae breeding sites, inside and outside homes, were reared to obtain adults for species identification. The Breteau Index and Container Index were then calculated for the *Aedes* spp. identified. Collection of adult mosquitoes was performed indoors with pyrethrum spray catches and outside with US Centers for Disease Control and Prevention (CDC) light trap and oral aspirators (for hand catches).

Epidemiologic study was conducted March 21–28, 2006. A retrospective cross-sectional study was performed on Toamasina’s population (≈200,000 inhabitants) by using a 2-stage cluster sampling (n = 378) at the neighborhood level. Every member of the household was interviewed with a standardized questionnaire based on personal history of sickness and travel. Data for children or those absent were given by present adults. Description of fever episodes and other clinical signs occurring after January 1, 2006, were collected. DLS is defined as a febrile illness and at least 2 other signs or symptoms: headache, joint pain, body pain, rash, and asthenia. Data were entered and analyzed by using EpiInfo (CDC, Atlanta, GA, USA).

**Figure 1:**
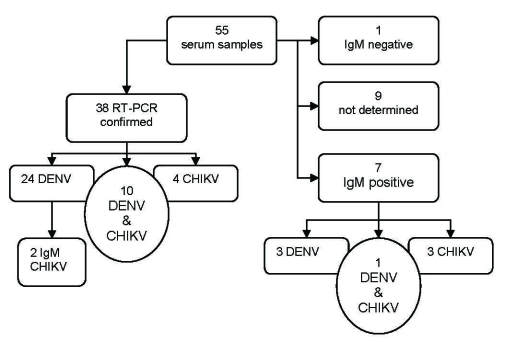
Laboratory diagnosis, dengue (DENV) and chikungunya (CHIKV) viruses, for 55 patients sampled, Madagascar, 2006. RT-PCR, reverse transcription–PCR; Ig, immunoglobulin.

DENV-1 or CHIKV was detected in 38 of 55 patients sampled. Co-infections were detected in 10 patients (Figure 1). Consequently, the outbreak was definitively attributed to DENV-1 and CHIKV.

*Aedes albopictus* was the unique DENV and CHIKV vector found. Only 6 adults of this mosquito species were caught. All of the positive larval breeding sites consisted of artificial containers (drums, buckets, coconut shells, discarded cans, tires, pots, and wet containers). Tires were the most important breeding sites identified (68%, n = 96). The Container and Breteau Indexes were 38.6 and 84.0, respectively. *Ae. albopictus* mosquito pools (n = 23) were tested by processing with CHIKV and DENV reverse transcription–PCRs. Five pools, including adults reared from larvae, were found to be CHIKV positive. However, no isolate was obtained from these pools by using AP61 cells.

A total of 4,242 residents (803 household units) in the 27 neighborhoods of Toamasina participated in the study. The mean age of participants was 24.9 years (95% confidence interval [CI] 24.4–25.4). The sex ratio (male:female) was 0.87. Most of the participants (n = 3,084; 72.7%) reported >1 previous episode of fever from January through March 2006. DLS was suspected in 2,863 (67.5%) residents, among whom 41 (1.4%) were hospitalized.

**Figure 2:**
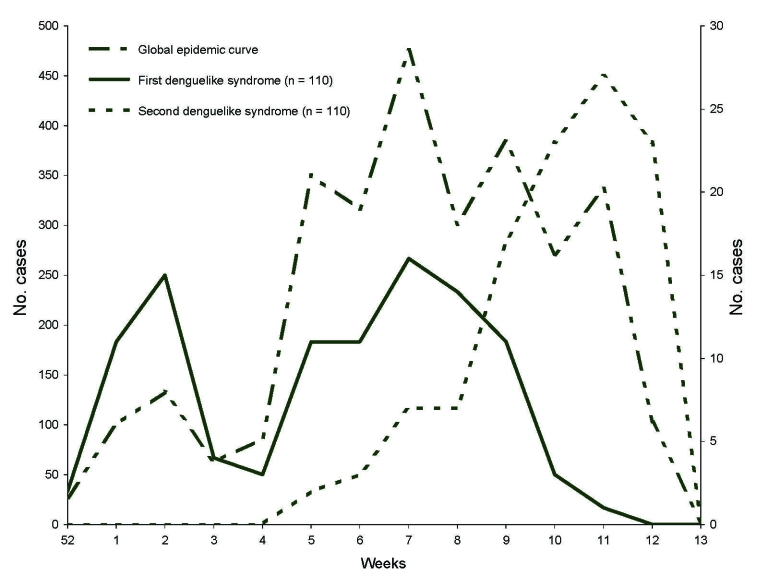
Outbreak curve of dengue and chikungunya fevers in Toamasina, January 1–March 28, 2006.

The estimated epidemic curve identified 2 outbreak periods. This distribution was emphasized by the report of a second episode of DLS by 110 patients. The peaks of the 2 periods were estimated in weeks 7 and 11 (Figure 2). The percentage of DLS in the studied population was high (40.2%–76.1%), regardless of age or sex. Rates of DLS did no differ in male and female patients. However, patients with DLS were older (mean age 26.8 years, 95% CI 26.2–27.4) than nonaffected persons (mean age 20.8 years, 95% CI 19.9–21.7; p<0.01). The most common features of the first episode of DLS (n = 110) were fever (100%), headache (96.4%), joint pain (79.1%), asthenia (76.4%), myalgia (76.4%), pruritus (40.0%), and rash (13.6%). When these 110 patients exhibited a second episode of DLS, the most common clinical features were fever (100%), headache (90.0%), myalgia (73.6%), joint pain (68.2%), and pruritus (28.2%).

## Conclusions

An outbreak caused by both CHIKV and DENV-1 occurred in Madagascar. The notification of CHIKV in the island occurred during a large epidemic that hit the southwestern islands of the Indian Ocean. The Malagasy CHIKV E1 partial nucleotide sequences were similar to those of the Indian Ocean outbreak (Réunion, Seychelle, Mayotte, Mauritius islands) and represented a distinct clade within the large east-central and South African phylogroup (*1*). DENV-1 was also circulating during this outbreak in the eastern part of the island. A few DENV-1 isolates were already obtained from patients visiting the northwestern coast of the island in early 2005. Molecular characterization of these isolates showed that they were similar to DENV-1 strains circulating in 2004 in Réunion and indicated their regional origin (H. Zeller, pers. comm.).

*Ae. albopictus* was the only urban vector of DENV or CHIKV detected during this outbreak. This species had already been reported in the eastern coast of Madagascar (*5*). Individual mosquitoes from this species, sampled in Antananarivo (in the highlands of Madagascar) in the 1970s and more recently in northern Madagascar, are highly susceptible to CHIKV and DENV (*6*–*8*). This situation, associated with a high larvae index and probably a nonimmune population, led to a high rate of transmission, as indicated by the high percentage of DENV-1 and CHIKV co-infection detected in the 55 patients tested (18%) and the high attack rate (67.5%).

The cocirculation of DENV-1 and CHIKV during the period may explain the 2 peaks observed in the epidemic curve. However, because the study was based on clinical observations alone, we could not measure the relative contribution for each peak. All age groups were affected. This result is likely due in part to the recent emergence of these viruses in Madagascar. However, children and particularly those <1 year of age might be less exposed to *Ae. albopictus* because they are often indoors and are fully covered. Underreporting could also be an explanation for this moderate prevalence in children.

During this epidemic, vector control measures (removal of tires from rooftops, information campaign, education of the community) were undertaken by the local authorities. After the outbreak, the Malagasy Ministry of Health implemented a sentinel surveillance system to monitor and control epidemic arboviral diseases. However, the emergence of this regional outbreak also demonstrates the need for coordinating surveillance systems in the islands in the southwestern Indian Ocean.
